# The Diagnostic Approach to Mitochondrial Disorders in Children in the Era of Next-Generation Sequencing: A 4-Year Cohort Study

**DOI:** 10.3390/jcm10153222

**Published:** 2021-07-22

**Authors:** Deborah Tolomeo, Daniele Orsucci, Claudia Nesti, Jacopo Baldacci, Roberta Battini, Claudio Bruno, Giorgia Bruno, Denise Cassandrini, Stefano Doccini, M. Alice Donati, Annarita Ferrari, Simona Fiori, Chiara Fiorillo, Renzo Guerrini, Francesco Mari, Martino Montomoli, Francesca Pochiero, Elena Procopio, Lucia Ruggiero, Simone Sampaolo, Federico Sicca, Chiara Ticci, Anna Rubegni, Filippo M. Santorelli

**Affiliations:** 1 Department of Molecular Medicine, IRCCS Stella Maris Foundation, 56128 Pisa, Italy; deborah.tolomeo01@gmail.com (D.T.); cla_nesti@yahoo.it (C.N.); jacopo.baldacci@hotmail.it (J.B.); dcassandrini@fsm.unipi.it (D.C.); stefanodoccini@gmail.com (S.D.); 2 Department of Neuroscience, Psychology, Drug Research and Child Health, University of Florence, 50139 Florence, Italy; 3 Unit of Neurology, San Luca Hospital, 55100 Lucca, Italy; orsuccid@gmail.com; 4 Department of Clinical and Experimental Medicine, University of Pisa, 56100 Pisa, Italy; roberta.battini@fsm.unipi.it; 5 Department of Clinical Neuroscience, IRCCS Fondazione Stella Maris, Calambrone, 56128 Pisa, Italy; annarita.ferrari@fsm.unipi.it (A.F.); simona.fiori@fsm.unipi.it (S.F.); federico.sicca@fsm.unipi.it (F.S.); chiara.ticci@fsm.unipi.it (C.T.); 6 Center of Translational and Experimental Myology, IRCCS Istituto Giannina Gaslini, 16147 Genoa, Italy; claudio2246@gmail.com (C.B.); chifiorillo@gmail.com (C.F.); 7 Department of Advanced Medical and Surgical Sciences, 2nd Division of Neurology, University of Campania “Luigi Vanvitelli”, 80131 Naples, Italy; giorgiabruno990@gmail.com (G.B.); simone.SAMPAOLO@unicampania.it (S.S.); 8 Metabolic Disease Unit, Meyer Children’s University Hospital, 50139 Florence, Italy; m.donati@meyer.it (M.A.D.); francesca.pochiero@meyer.it (F.P.); elena.procopio@meyer.it (E.P.); 9 Neurology Unit and Neurogenetics Laboratories, Meyer Children Hospital, 50139 Florence, Italy; renzo.guerrini@meyer.it (R.G.); francesco.mari@meyer.it (F.M.); martino.montomoli@meyer.it (M.M.); 10 Department of Neurosciences, Reproductive and Odontostomatological Sciences, University Federico II of Naples, 80131 Naples, Italy; ruggilucia@gmail.com

**Keywords:** diagnostic approach, mitochondrial disorders, next-generation sequencing, mtDNA, nDNA, muscle biopsy, MRI, basal ganglia

## Abstract

Mitochondrial diseases (MDs) are a large group of genetically determined multisystem disorders, characterized by extreme phenotypic heterogeneity, attributable in part to the dual genomic control (nuclear and mitochondrial DNA) of the mitochondrial proteome. Advances in next-generation sequencing technologies over the past two decades have presented clinicians with a challenge: to select the candidate disease-causing variants among the huge number of data provided. Unfortunately, the clinical tools available to support genetic interpretations still lack specificity and sensitivity. For this reason, the diagnosis of MDs continues to be difficult, with the new “genotype first” approach still failing to diagnose a large group of patients. With the aim of investigating possible relationships between clinical and/or biochemical phenotypes and definitive molecular diagnoses, we performed a retrospective multicenter study of 111 pediatric patients with clinical suspicion of MD. In this cohort, the strongest predictor of a molecular (in particular an mtDNA-related) diagnosis of MD was neuroimaging evidence of basal ganglia (BG) involvement. Regression analysis confirmed that normal BG imaging predicted negative genetic studies for MD. Psychomotor regression was confirmed as an independent predictor of a definitive diagnosis of MD. The findings of this study corroborate previous data supporting a role for neuroimaging in the diagnostic approach to MDs and reinforce the idea that mtDNA sequencing should be considered for first-line testing, at least in specific groups of children.

## 1. Introduction

Mitochondrial diseases (MDs) are a large and heterogeneous group of genetically determined multisystem disorders mainly related to the oxidative phosphorylation (OXPHOS) system [1,2]. Collectively, they are caused by the most common inborn errors of metabolism [3]. MDs occur in a large section of the lifespan (from the neonatal period to late adulthood), with an incidence of about 1.6/5000 [4]; in the pediatric population, the incidence is 0.5–1.5/100,000 [5,6]. In the majority of cases, onset occurs in infancy or early childhood and is associated with high morbidity and mortality [7,8,9]. The significant clinical variability of MDs is related to the extreme genetic heterogeneity of these conditions, linked to mutations in both nuclear (nDNA) and mitochondrial DNA (mtDNA). The unpredictable role of tissue heteroplasmy in cases with defects in the mitochondrial genome, and that of genetic modifiers [10], may further influence the phenotype and the extent of intrafamilial variability.

Over the past three decades, more than 250 mtDNA mutations have been identified, and the use of massive gene sequencing technologies such as next-generation sequencing (NGS) is now enabling the identification of a growing number of new nDNA mutations [5,6], found mainly in the pediatric population [11]. NGS is not easy to use in the clinical setting on account of the massive number of data it yields: in patients with MDs, it is difficult to select candidate variants and interpret their disease-causing role. To address these difficulties, several studies have proposed combining genetic and functional investigations in situations where rapid and more effective disease management is required, as in cases of pyruvate dehydrogenase deficiency and Leigh syndrome (LS), or where a tissue-specific condition is suspected (e.g., a pure myopathy related to mtDNA mutations) [12,13]. The correct use of biochemical testing and muscle histology studies, in combination with neuroimaging, has proved helpful in genotype-imaging-phenotype correlations [14], albeit with limitations in terms of specificity and sensitivity [15].

However, there is still a shortage of reviews assessing whether clinical, imaging, and muscle biopsy studies performed in the clinical practice setting might impact the molecular diagnostic yield in MDs [16,17].

We retrospectively evaluated the molecular diagnostic yield obtained with the use of an NGS gene panel in 111 unrelated pediatric patients referred to our center to undergo molecular and functional investigations due to clinical suspicion of MD. We attempted to identify single predictors of a molecular MD diagnosis.

## 2. Materials and Methods

All the procedures reported in this study were performed in compliance with the 1975 Helsinki Declaration and with written parental informed consent for muscle biopsy and molecular analyses. 

### 2.1. Clinical Data

In this real-life clinical setting study, we retrospectively evaluated data in 111 consecutive cases of suspected MD referred to us from multiple Italian centers over a 48-month period (January 2016–December 2019) in order to perform histological, immunohistochemical, biochemical, or molecular analyses. The majority of patients presented a complex clinical phenotype different from a rapidly recognizable mitochondrial syndrome [18]. In our dataset, dichotomous (“yes or no”) variables are used to report the presence/absence of clinical data and neuroimaging features (a few examples of the data collected are provided in Table S1). 

### 2.2. Histochemical and Spectrophotometric Investigations in Muscle Biopsy

Diagnostic muscle biopsy was performed in all patients according to standard procedures and using routine histochemical stains [19]. Prior to molecular investigations, three expert co-authors (C.F., A.R., and S.S.) blindly revised the patients’ histological features. Spectrophotometric measurements of respiratory chain (RC) enzyme complex activities and citrate synthase (CS)—a measure of mitochondrial mass—were performed using reported methodologies in a single laboratory [20]. Data of RC complex activities were reported after correction to the activity of CS and were classified as mild (>50%), moderate (25–50%), and severe (<25%) based on the residual enzyme activity compared to the average of the results obtained in control samples.

### 2.3. Molecular Studies

Total genomic DNA was purified from available tissues (skeletal muscle, blood, skin fibroblasts, and urine) using an automated extractor (Magpurix, Zinexts Life Science Corporation, Taipei, Taiwan). Sequencing of the whole mitochondrial genome was performed in 82/111 subjects using Nextera XT technology (Illumina, San Diego, CA, USA) following the protocol “Human mtDNA Genome”; libraries were sequenced with paired-end 2 × 150 reads on a MiSeq desktop sequencer (Illumina, San Diego, CA, USA). This approach allowed us to sequence the mitochondrial genome at a high depth of coverage (>5000× on average). The clinical significance of the identified mtDNA variants was assessed based on mitochondrial variation databases (Mitomap, www.mitomap.org, accessed on 8 July 2021) and the bioinformatic tools commonly used in our NHS-certified mitochondrial laboratory, such as MitImpact (https://mitimpact.css-mendel.it, accessed on 8 July 2021) and HmtVar (www.hmtvar.uniba.it, accessed on 8 July 2021). In particular, the aligned reads were analyzed for variant calling and heteroplasmy level detection with the Ingenuity Variant Analysis tool (Qiagen, Hilden, Germany). Parameters were the following: minimum basecall quality score >30, analysis threshold at 1%, interpretation threshold at 1%, and minimum read count at 1000× (needed for assessing heteroplasmy). Capillary sequencing confirmed the variants identified in patients and demonstrated segregation in available maternal relatives. Relative read count indicated by Integrative Genomics Viewer software was used to assess levels of heteroplasmy in specific mtDNA variants in tissues and individuals by calculating the percentages of the four nucleotides detected in the same position.

The search for mtDNA deletions (single or multiple) in muscle DNA was performed using a long-template PCR amplification method, amplifying different mtDNA regions using various sets of primers adopting Ranger DNA Polymerase (Meridian Lifescience, Memphis, TN, USA), specifically designed to amplify long genomic DNA templates. A reported qPCR analysis was used to test for the presence of the so-called “common” deletion [21] or to assess gene copy number and define the extent of mtDNA depletion [22]. One limitation is that the method cannot indicate deletions below about 100 bp.

Using methods described elsewhere [23], we analyzed in 27 children the “MitoExome”, a customized multigene panel targeting the coding regions of 1172 genes associated with mitochondrial pathways [24]. Briefly, the panel was designed with the NimbleGen Design software (Roche NimbleGen Inc., Pleasanton, CA, USA), and target enrichment and amplification were performed following the SeqCap EZ HyperCap Library protocol (Roche, Madison, WI, USA), adapted for a MiSeq desktop scanner (Illumina, San Diego, CA, USA). An in-house bioinformatic pipeline, based on the Ingenuity Variant Analysis bioinformatic suite (IVA, Qiagen, Germany), and the web tool Annovar (http://wannovar.usc.edu/, accessed on 8 July 2021) were used to filter variants and prioritize genes/alleles of interest. Data were also prioritized based on minor allele frequencies (defined in gnomAD 2.1, https://gnomad.broadinstitute.org/, accessed on 8 July 2021) and CADD scores (CADD, Combined Annotation Dependent Depletion; https://cadd.gs.washington.edu/snv, accessed on 8 July 2021). Missense variants were also systematically evaluated for the functional consequences in silico using Polyphen2 (http://genetics.bwh.harvard.edu/pph2/, accessed on 8 July 2021) and SIFT (Sorting Intolerant From Tolerant) (http://sift.jcvi.org/, accessed on 8 July 2021) predictions. To evaluate possible deleterious effects of synonymous and missense variants on splicing, we used the Human Splicing Finder web tool (Genomsis, Marseille, France). All sequenced samples had at least 98% of target covered 20× or more.

### 2.4. Statistical Analyses

Statistical analyses were performed using the Jamovi v. 1.1.9.0 statistical suite (www.jamovi.org, accessed on 8 July 2021) and R 4.0.3 × 64. Statistical significance (Jamovi, Sydney, Australia) was set at a two-tailed *p* of <0.05. Fisher’s exact test was used for categorical associations. Continuous variables were compared by unpaired two-tailed Student’s *t*-test (or by Mann–Whitney test when the data were not normally distributed). Bonferroni’s correction for multiple tests was applied where appropriate. In our study, a “non-significant trend” was defined by a *p*-value not showing formal statistical significance after Bonferroni’s correction. Multiple logistic regressions were applied to test the combinations of significant variables.

## 3. Results

### 3.1. Patient Demographics and Clinical Presentations

**Figure 1:**
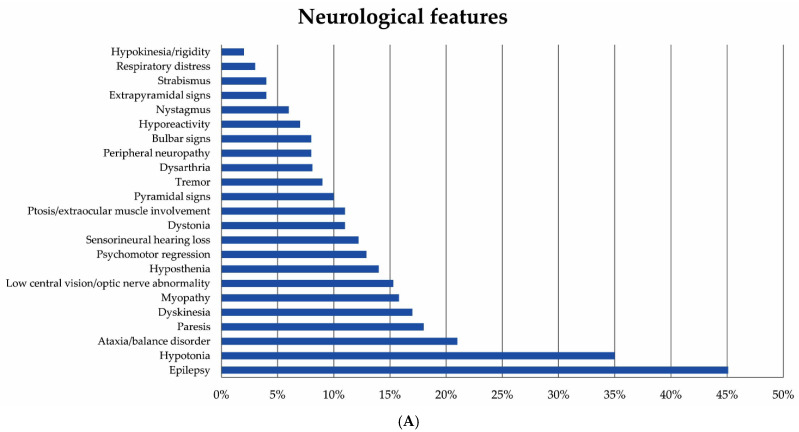

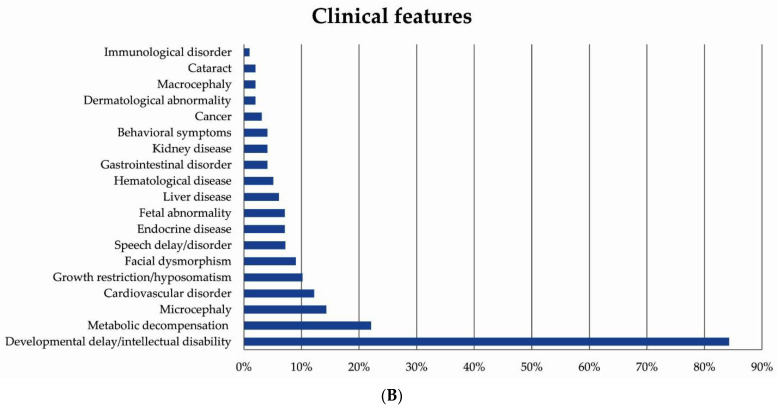
(**A**) The prevalence rates of the main neurological features displayed by the patients at the time of this study. (**B**) The prevalence rates of the general clinical features displayed by the patients at the time of referral to us.

In the 111 patients (63 boys and 48 girls), the onset of the symptoms occurred in infancy in 42 cases, in childhood in 64, and in adolescence (between 13 and 18 years of age) in two. In three patients, we could not ascertain the precise age at onset. On referral to us, full neurological information was provided in most cases (106/111, 95%). At the time of our study, the most frequent neurological features (Figure 1A) were epilepsy (referred in 46/102 children), hypotonia (35/100), and ataxia (21/100). Figure 1B illustrates the main non-neurological clinical features presented by the study group.

Neurometabolic blood biomarkers were analyzed in 44 patients. We recorded elevated lactic acid levels in 68.2% of cases, Krebs cycle intermediate metabolites in 52.3%, and high serum levels of alanine in 38.6%. 

### 3.2. Neuroimaging Features

**Figure 2:**
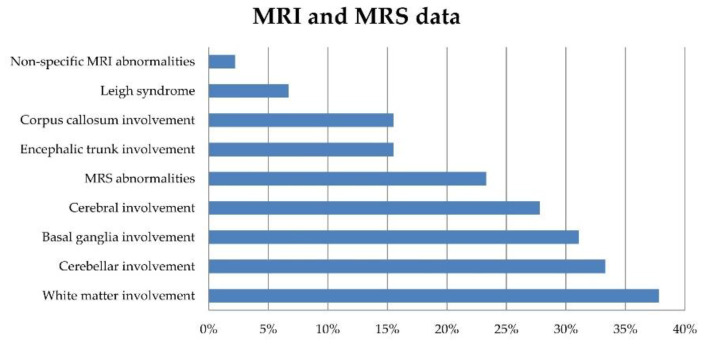
Prevalence rates of brain MRI and MRS features in 90 patients at the time of their referral to us.

Neuroimaging data were available in 90 patients. The most frequent neuroradiological presentations included extensive white matter changes (37.8%), cerebellar atrophy (33.3%), basal ganglia (BG) involvement (31.1%), cerebral atrophy (27.8%), and brainstem and corpus callosum involvement (each present in 15.5% of the patients). Brain MRI characteristics meeting the accepted criteria for LS were found in a few patients (6.7%). Magnetic resonance spectroscopy (MRS) abnormalities were reported in 21/90 patients (23.3%) (Figure 2).

### 3.3. Skeletal Muscle Biopsy: Histological and Biochemical Analyses

**Figure 3:**
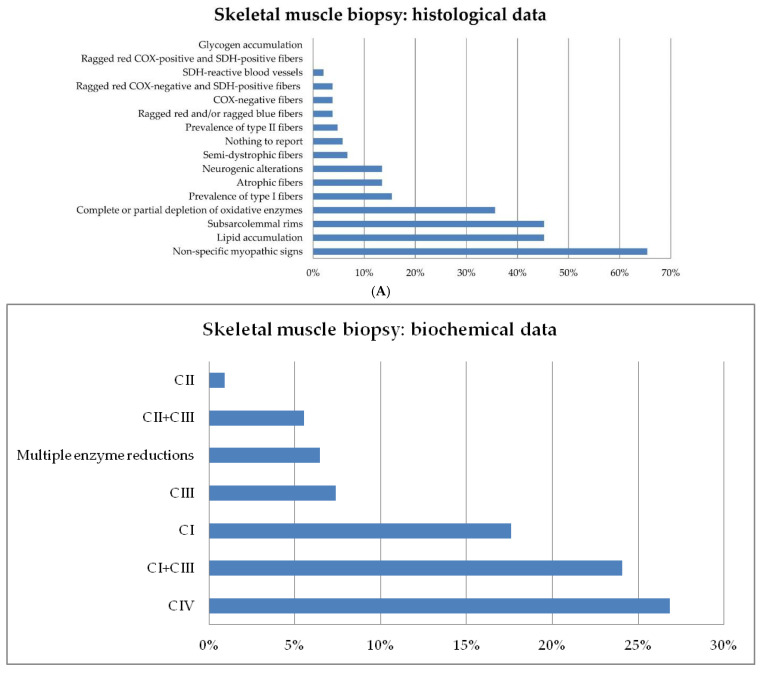
(**A**) Prevalence rates of morphological features in the 104 patients who underwent histological analysis of skeletal muscle. (**B**) Relative frequencies of respiratory chain enzyme deficiencies.

On referral to our centers, muscle biopsy histology data were available in 104 patients. In the other seven cases, the small size of muscle samples prevented their examination. These data show the presence of non-specific myopathic signs in 68/104, lipid accumulation in 47/104, subsarcolemmal rims in 47/104, and depleted oxidative enzyme stains in 37 patients (Figure 3A). 

Data on RC enzyme activity in muscle biopsies were available in 108/111 patients, while the other three samples were of low technical quality and could not be examined. Low activity of complex IV (denoting moderate or severe enzyme deficiency) was the most common result (29/108 patients). The analysis also shows reduced activity (corresponding to mild or moderate deficiency) of complexes I + III (CI + CIII, *n* = 26), complex I (CI, *n* = 19), complex III (CIII, *n* = 8), complexes II + III (CII + CIII, *n* = 6), and complex II (CII) in a single patient who harbored a homozygous mutation in *PITRM1*. Multiple enzyme reductions were observed in seven patients (Figure 3B). In most cases (82/108) CS was within the normal range. 

### 3.4. Molecular Genetics

**Figure 4:**
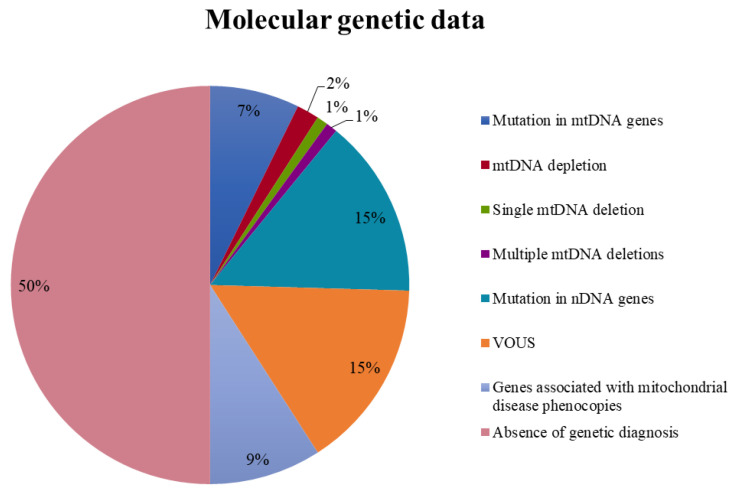
Molecular genetic results in this study (expressed as percentages of patients). VOUS: variants of unknown significance in nDNA and mtDNA genes.

A definitive molecular diagnosis was reached in 28 patients; of these, 16 presented mutations in nDNA-encoded mitochondrial genes (fully reported in Table S2A), and eight harbored point mutations in mtDNA (detailed in Table S2B). Two children harbored deletions (one single and one multiple) in the mitochondrial genome, while another two harbored mtDNA depletion in skeletal muscle. In the latter, we were unable to clarify the underlying molecular mechanism (Figure 4). Subsequently, in 10 patients, pathogenic or likely pathogenic variants in genes associated with clinical conditions mimicking MD were found by performing WES analysis (namely *GFAP*, *GABRA1*, *GMPPB*, *PMM2*, *MECP2*, *FOXG1*, *CACNA1*, *PRUNE1*, and *PRRT2*, the latter detected in two patients). 

Variants of unknown significance (VOUS) in nDNA and mtDNA genes were found in 17 patients (see Table S3). Variants were defined VOUS in the presence of incomplete segregation (i.e., lack of parental samples to allow phasing of two variants in cis/trans) when perfect correlation with the reported clinical presentation was lacking or when a single deleterious variant occurred in an autosomal recessive gene in the absence of functional studies. In 55 patients, no genetic diagnosis was reached during this study.

### 3.5. Predicting a Molecular Diagnosis of MD

We sought to identify factors linked to a definitive molecular diagnosis of MD. Age at onset and gender did not differ between the patients with a definitive molecular diagnosis (*n* = 28) and the remaining patients (*n* = 83). On investigation of metabolic and clinical features, we can speculate on a trend towards association with a definitive diagnosis of MD only for psychomotor regression (*p* = 0.0045). Neither biochemical nor histological data seemed to predict molecularly confirmed MD, although the presence of both COX-negative and SDH-positive ragged red fibers showed a trend in this direction (*p* = 0.0038). When we analyzed overall MRI features, BG involvement was significantly associated with a definitive diagnosis of MD (*p* = 0.0001), being found in 64.0% of the confirmed patients versus 19.4% of the remaining ones. In the 16 patients with an nDNA mutation, developmental delay/intellectual disability (DD/ID) was the main clinical feature (11/16), followed by epilepsy (8/16) and hypotonia (7/16). BG involvement was found in half of these patients (including a single case with LS), whereas MRS alterations were found in six. Neurometabolic alterations were found in 8/16, mainly high serum levels of alanine. Nonspecific myopathic changes and reduced RC complexes I + II and IV activities were more common in this cohort. However, there was no significant association between demographic, clinical, metabolic, imaging, and muscle studies and a definitive diagnosis of nDNA-associated MD. In the eight patients with mtDNA mutations, DD/ID was again the main clinical feature. Epilepsy, on the other hand, was more common in patients without mtDNA variants (*p* = 0.039). The presence of both COX-negative and SDH-positive ragged red/blue fibers (*p* = 0.037) showed a trend towards significance in the patients harboring mtDNA mutations. BG involvement (6/8) was correlated with a definitive diagnosis of an mtDNA-associated disease *(p* = 0.008) (Table S2B).

A multiple logistic regression, with “Y” defined as a diagnosis of definitive MD versus another or no diagnosis, was applied to test a predictive equation. “X” was defined by the following variables: DD/ID, epilepsy, COX-negative and SDH-positive ragged red/blue fibers, and BG involvement. The model had a high level of significance (*p* = 0.0000092). The finding of BG abnormalities on neuroimaging (*p* = 0.00076; 95% confidence interval, odds ratio 0.03–0.39) and the presence of DD/ID (*p* = 0.0022; 95% confidence interval, odds ratio 0.04–0.77) were the sole independent predictors of a confirmed MD diagnosis (Table S4). This model was particularly robust when considering cases with nDNA mutations (*p* = 0.0034; 95% confidence interval, odds ratio 0.02–0.45); in contrast, a model in which “Y” was an mtDNA-related diagnosis did not reach significance, likely due to the low numbers. None of the other clinical, neuroradiological, histological, and biochemical features were found to predict MD.

## 4. Discussion

In recent years, sophisticated NGS molecular testing tools (gene panels and exome and genome sequencing) have entered clinical practice, allowing the identification of new genes, and facilitating molecular studies in genetically heterogeneous conditions such as MDs. With such new tools at hand, some investigators have attempted to use shortcuts in molecular studies, preferring a reverse phenotyping (i.e., “genotype-first”) approach [12] rather than a more classical and logical diagnostic workup, where clinical and imaging studies are combined with studies of skeletal muscle (“biopsy-first” approach). Even though NGS techniques lead to a higher diagnostic yield (40–60% depending on the cohort) [25,26,27], they still have some major limitations, and it remains unclear how to counsel general pediatricians on which approach is more robust in clinical practice.

Our study is the first attempt to define features potentially influencing a definitive diagnosis of MD, and it allows us to highlight a series of aspects of the diagnostic process. First, we corroborated the notion that mutations in nDNA-related genes are more common in children than mutations in mtDNA [28]. Accordingly, a massive study of nDNA genes—we used a MitoExome panel—would seem to be appropriate when faced with a potential new pediatric case of MD. Second, psychomotor regression and seizures, either alone or in combination, emerged as “red flags” potentially able to shed light on a case of clinically suspected MD [29]. This is a further element suggesting that the occurrence of epilepsy in children should be investigated carefully for the possible presence of an OXPHOS disorder [30,31,32] and properly investigated at the genetic level. In our cohort, epilepsy was more frequent in patients with an nDNA-related disorder (50%) than in those harboring mtDNA mutations (11.1%), as we have previously shown [33]. Third, however, MD-like clinical manifestations in children can remain vague. The phenotypes of 9% of our patients were phenocopies, as these patients harbored mutations in genes not directly responsible for a primary mitochondrial dysfunction [15]. Only the integration of metabolic investigations, neuroimaging, muscle biopsy, and ultimately genetic testing allowed us to reach the final diagnosis in this group. Fourth, we demonstrated that careful imaging assessment is crucial in children with a suspected diagnosis of MD and that BG involvement is the single independent predictor of a definitive diagnosis. This finding was not particularly surprising given that deep brain structures, because of their high metabolic activity, are rich in mitochondria and therefore more vulnerable to metabolic and systemic disorders [34,35]. Although the presence of bilateral and symmetrical BG involvement suggestive of LS was uncommon in our cohort (observed in four children only), this finding nevertheless further reinforces the importance of the BG in MDs as already reported [36]. It is also interesting to note that mutations in subunits of RC complexes I and IV have already been described in association with frequent BG involvement [37,38], and it is worth considering that this finding could help in prioritizing gene testing.

We observed that neither neurometabolic nor biochemical measurements of RC complex enzymes appeared able to drive a clear diagnosis. Conversely, the second most important paraclinical tool in predicting a definitive diagnosis of MD in our cohort was the muscle biopsy histology, even though the data did not reach statistical significance. Given the nature of our sample, in most of our patients, the procedure took place very early in life (infancy or early childhood), and the vague or poorly defined results such as non-specific myopathic signs, lipid accumulation, and subsarcolemmal rims obtained in some cases may therefore be due to relatively small biopsy sample sizes or to the fact that the procedure was performed early in life. However, our findings are in keeping with those reported in a larger pediatric cohort where ragged red fibers and/or COX-negative fibers correlated well with mtDNA mutations in 90% of the biopsies [39]. Overall, our data seem to highlight the importance of imaging and skeletal muscle studies in new pediatric cases with a diagnosis compatible with MD. Even if such studies fail to show abnormalities suggestive of MD, this outcome, too, might allow alternative diagnoses to be considered before embarking on further, more time-consuming investigations.

This work presents several limitations. First, the type and timing of the molecular analyses, linked to the retrospective and multicenter nature of the study, might have influenced the diagnostic yield; indeed, a large set of cases remained unsolved even after full MitoExome studies. It should, however, be said that inclusion of the VOUS cases would have given us a diagnostic yield of 41%, a figure corresponding to what has recently been observed in exome studies [27,40]. Even more recently, whole-genome (WGS) approaches have contributed to the detection of deep intronic variants and deletions, and a 3′UTR duplication, as well as the identification of novel disease genes (*COX6A1, TIMMDC1*, and *COQ5*) [41], none of which were explored in our cases. The true contribution of WGS to the diagnosis of mitochondrial diseases is unclear because of its limited use in clinical practice, but it seems likely to give a higher diagnostic yield than exome analysis, showing rates similar to those it has given in other rare diseases (about 40–50%) [42].

Another important limitation relates to the incompleteness of the extra-neurological information provided at the time of the study. Most cases were referred to us from neuropediatric services, and the very young age of most of the cases might, in some instances, have limited our full appraisal of their multisystem syndrome.

## 5. Conclusions

The present study, although retrospective, evaluated a range of features in a large cohort of children with possible MD. MRI evidence of BG involvement emerged as the strongest predictor of a definitive diagnosis of MD. From a clinical perspective, psychomotor regression was also confirmed as an independent predictor of a definitive diagnosis of MD. Our data might help future prospective studies in multicenter settings.

## Data Availability

Data can be obtained by the corresponding authors upon reasonable request.

## Supplementary Materials

The following are available online at https://www.mdpi.com/article/10.3390/jcm10153222/s1, Table S1: A few examples of clinical, neuroimaging, histopathological, and biochemical features data collection in patients with final genetic diagnosis of MDs. Table S2A: Nuclear DNA mutations identified in our patients. Table S2B: Mitochondrial DNA mutations identified in our patients. Table S3: Variants of unknown significance (VOUS) in nDNA and mtDNA. Table S4: Multiple logistic regressions. The 95% confidence intervals for the odds ratios of the significant variables (psychomotor regression and, especially, basal ganglia involvement) are reported in the main text.

## Abbreviations

COX = cytochrome *c* oxidase; SDH = succinate dehydrogenase; NADH = nicotinamide adenine dinucleotide; CI = complex I; CII= complex II; CIII = complex III; CS = citrate synthase; n.a. = data not available. DD/ID = developmental delay/intellectual disability; WM = white matter; BG =basal ganglia; MRS = magnetic resonance spectroscopy; RRF: ragged red fibers.
