# Supplementary material for: The Diagnostic Approach to Mitochondrial Disorders in Children in the Era of Next-Generation Sequencing: A 4-Year Cohort Study
Source: J Clin Med. 2021 Jul 22;10(15):3222. doi: 10.3390/jcm10153222 (PMC8348083; doi:10.3390/jcm10153222)
Supplement: Supplementary file 1 [file jcm-10-03222-s001.zip › jcm-1250779-conversion/Table S1.pdf]

Gene	*MTTL1*	*CLPP*	*SURF1*
**Age at onset**	Childhood	Infancy	Infancy
**Gender**	F	F	M
**Metabolic decompensation**	Yes	No	No
**Endocrine disease**	No	No	No
**Growth restriction/hyposomatism**	No	Yes	Yes
**Cataract**	No	No	No
**Low central vision/optic nerve abnormality**	No	No	No
**Sensorineural hearing loss**	No	Yes	No
**Fetal abnormality**	No	Yes	No
**Cardiovascular disorder**	No	Yes	No
**Liver disease**	No	No	No
**Epilepsy**	No	No	No
**Psychomotor regression**	No	No	Yes
**Facial dysmorphism**	No	No	Yes
**Hyposthenia**	Yes	Yes	No
**Hypotonia**	No	Yes	Yes
**Myopathy**	Yes	No	No
**Ataxia/balance disorder**	No	Yes	No
**Dysarthria**	No	No	Yes
**Dystonia**	No	No	No
**Hypokinesia/rigidity**	No	No	No
**Dyskinesia**	No	No	Yes
**Pyramidal signs**	No	No	No

**Extrapyramidal signs**	No	No	No
**Tremor**	No	No	Yes
**Peripheral neuropathy**	No	No	Yes
**Strabismus**	No	No	No
**Ptosis/extraocular muscle involvement**	No	No	No
**Nystagmus**	No	No	Yes
**Respiratory distress**	No	Yes	No
**Hyporeactivity**	No	No	No
**Bulbar signs**	No	No	No
**Developmental delay/intellectual disability**	Yes	Yes	Yes
**Leigh syndrome**	No	No	Yes
**Cerebral involvement**	Yes	No	Yes
**Cerebellar involvement**	No	Yes	Yes
**White matter involvement**	Yes	Yes	No
**Basal ganglia involvement**	Yes	No	Yes
**Encephalic trunk involvement**	No	Yes	Yes
**Corpus callosum involvement**	No	Yes	No
**MRS abnormalities**	n.a.	n.a.	Yes
**Neurometabolic screening**	n.a.	Yes	n.a.
**Elevated lactic acid levels**	n.a.	No	n.a.
**Elevated alanine levels**	n.a.	Yes	n.a.
**Elevated levels of other metabolites**	n.a.	No	n.a.
**Atrophic fibers**	Yes	No	No
**Semi-dystrophic fibers**	Yes	No	No

**Ragged red and/or ragged blue fibers**	Yes	No	No
**COX-negative fibers**	Yes	Yes	Yes
**Complete or partial depletion of oxidative enzymes**	Yes	No	Yes
**Ragged red COX-negative and SDH-positive fibers**	Yes	Yes	Yes
**Ragged red COX-positive and SDH-positive fibers**	No	No	No
**Lipid accumulation**	Yes	Yes	No
**Non-specific myopathic signs**	No	Yes	No
**Neurogenic changes**	No	No	No
**Glycogen accumulation**	No	No	No
**Subsarcolemmal rims**	Yes	Yes	No
**SDH-reactive blood vessels**	No	No	No
**Prevalence of type I fibers**	No	No	No
**Prevalence of type II fibers**	No	No	No
**NADH ubiquinone oxidoreductase**	Severe reduction	Within normal range	Within normal range
**NADH dehydrogenase**	Moderate reduction	Within normal range	Within normal range
**SDH**	Within normal range	Within normal range	Within normal range
**NADH-cytochrome c reductase (CI+CIII)**	Severe reduction	Severe reduction	Severe reduction
**Succinate-cytochrome c reductase (CII+CIII)**	Moderate reduction	Severe reduction	Within normal range
**COX**	Severe reduction	Moderate reduction	Severe reduction
**Citrate synthase**	Increase	Within normal range	Increase
