# Supplementary material for: The Diagnostic Approach to Mitochondrial Disorders in Children in the Era of Next-Generation Sequencing: A 4-Year Cohort Study
Source: J Clin Med. 2021 Jul 22;10(15):3222. doi: 10.3390/jcm10153222 (PMC8348083; doi:10.3390/jcm10153222)
Supplement: Supplementary file 1 [file jcm-10-03222-s001.zip › jcm-1250779-conversion/Table S2A.pdf]

Age at onset	Gender	Clinical features	Neuroradiological features	Neurometabolic screening in blood	Skeletal muscle biopsy: histological analysis	Skeletal muscle biopsy: biochemical analysis	Gene	Mutations
Childhood	M	Epilepsy, hypotonia, dystonia, dyskinesia, paresis	Cerebral involvement	Acid lactic increase	Insufficient material	NADH moderate reduction;<br>SDH mild reduction;<br>CI+III moderate reduction;<br>CII+III moderate reduction;<br>COX moderate reduction	*NARS2*	c.716G>T (p.Gly239Val)/<br>c.749G>A (p.Arg250Gln)
Childhood	F	Psychomotor regression, DD/ID, hypotonia, dyskinesia	WM, BG involvement, MRS alterations	Alanine and other metabolites increase	Complete or partial depletion of oxidative enzymes, lipid accumulation, subsarcolemmal rims, non-specific myopathic signs	CI+CIII severe reduction;<br>CII+CIII mild reduction;<br>COX moderate reduction;<br>CS increase	*SUCLA2*	c.850C>T (p.Arg284Cys)/<br>c.968A>G (p.Asn323Ser)
Childhood	F	Low central vision/optic nerves abnormalities, hypoacusia, gastrointestinal and liver disorder, paresis	Cerebral involvement	n.a.	Complete or partial depletion of oxidative enzymes	CI: moderate reduction;<br>COX: mild reduction	*KARS*	c.815T>G/(p.Phe272Cys)/<br>c.1570T>C(p.Cys524Arg)
Infancy	M	Speech delay/disorder, epilepsy, ataxia/balance disorder, strabismus, DD/ID	Cerebellar and BG involvement, MRS alterations	Within normal limits	Non-specific myopathic signs, subsarcolemmal rims	SDH mild reduction;<br>CI+CIII severe reduction	*PITRM1*	c.2239dupG (p.Val747Glyfs*31)<br>(maternal uniparental disomy)
Infancy	F	Epilepsy, hypotonia, pyramidal and	Cerebellar, WM, BG and Corpus callosum	Within normal limits	Complete or partial depletion of oxidative enzymes,	NADH mild reduction;<br>CI+CIII severe reduction	*VARS2*	c.1100C>T (p.Thr367Ile)/<br>c.1100C>T (p.Thr367Ile)

		extrapyramidal signs, DD/ID	involvement, MRS alterations		subsarcolemmal rims, SDH-reactive blood vessels			
Childhood	F	Epilepsy, ataxia/balance disorder, dysarthria, paresis, extrapyramidal signs, DD/ID	Cerebellar involvement	Within normal limits	Non-specific myopathic signs, neurogenic alterations	Within normal limits	*COQ4*	c.284G>A (p.Gly95Asp)/<br>c.305G>A (p.Arg102His)
Infancy	M	Metabolic decompensation, cataract, hypoacusia, liver disorder	n.a.	Other metabolites increase	Complete or partial depletion of oxidative enzymes, non-specific myopathic signs	COX moderate reduction	*CLPB*	c.1078+2T>G/<br>c.1249C>T (p.Arg417*)
Childhood	M	Speech delay/disorder, low central vision/optic nerves abnormalities, psychomotor regression, hypotonia, nistagmus, DD/ID	Cerebellar, BG involvement, MRS alterations	n.a.	Non-specific myopathic signs	Within normal limits	*PLA2G6*	c.716T>C (p.Leu239Pro)/<br>c.2370T>G (p.Tyr790*)
Childhood	F	Low central vision/optic nerves abnormalities, epilepsy, psychomotor regression, ataxia/balance disorder, dystonia, peripheric neuropathy,	Cerebellar, WM and BG involvement	Alanine and other metabolites increase	Non-specific myopathic signs, lipid accumulation, subsarcolemmal rims	CI+CIII severe reduction	*RARS2*	c.1037C>T (p.Thr346Ile)/<br>c.517G>A (p.Asp173Asn)

		DD/ID						
Infancy	M	Cardiovascular disorder, epilepsy, hypotonia	WM, BG involvement; MRS alterations	Alanine increase	Within normal limits	CI moderate reduction SDH mild reduction CI+CIII severe reduction; CII+CIII severe reduction COX severe reduction	*NDUFA10*	c.161_162delCA (p.Thr54Argfs*33)/ c.296G>A (p.Gly99Glu)
Infancy	F	Growth restriction/hyposomatism, hypoacusia, fetal alterations, cardiovascular disorder, hypostenia, hypotonia, ataxia/balance disorder, respiratory distress, DD/ID	Cerebellar, WM, encephalic trunk and corpus callosum involvement	Alanine increase	RRF COX-negative and SDH-positive, lipid accumulation, subsarcolemmal rims, non-specific myopathic signs	CI+CIII severe reduction; CII+CIII mild reduction COX moderate reduction	*CLPP*	c.425C>T (p.Pro142Leu)/ c.425C>T (p.Pro142Leu)
Infancy	M	Growth restriction/hyposomatism, psychomotor regression, dysmorphic features, hypotonia, dysarthria, dyskinesia, tremor, peripheral neuropathy, nistagmus, DD/ID	LS, Cerebral, cerebellar, BG, encephalic trunk involvement; MRS alterations	n.a.	Complete or partial depletion of oxidative enzymes, RRF COX-negative and SDH-positive	CI+CIII severe reduction; COX severe reduction CS increase	*SURF1*	c.240+1G>T/ c.870insT (p.Lys291*)
Childhood	M	Ataxia/balance disorder	BG involvement	Acid lactic and other metabolites	Lipid accumulation and subsarcolemmal rims	Within normal limits	*AFG3L2*	c.2167G>A (p.Val723Met)/

				increase				c.634dupG (p.Val212fs*4)
Childhood	F	Low central vision/optic nerves abnormalities, epilepsy, psychomotor regression, paresis, tremor, strabismus, bulbar signs, DD/ID	WM involvement	Acid lactic, alanine and other metabolites increase	Complete or partial depletion of oxidative enzymes, prevalence of type I fibers	Within normal limits	*SCO2*	c.281T>C (p.Leu94Pro)/<br>c.281T>C (p.Leu94Pro)
Childhood	M	Behavioral disorder, epilepsy, DD/ID	Within normal limits	n.a.	Non-specific myopathic signs	Within normal limits	*POLG*	c.752C>T (p.T251I)/<br>c.1760C>T (p.P587L)
Childhood	M	Low central vision/optic nerves abnormalities, hypoacusia, ataxia/balance disorder, ptosis/ extraocular muscle involvement, DD/ID	n.a.	n.a.	Complete or partial depletion of oxidative enzymes, lipid accumulation and subsarcolemmal rims	CI+CIII mild reduction; CS increase	*RTN4IP1*	c.308G>A (p.Arg103His)/<br>c.308G>A (p.Arg103His)
