# Supplementary material for: The Diagnostic Approach to Mitochondrial Disorders in Children in the Era of Next-Generation Sequencing: A 4-Year Cohort Study
Source: J Clin Med. 2021 Jul 22;10(15):3222. doi: 10.3390/jcm10153222 (PMC8348083; doi:10.3390/jcm10153222)
Supplement: Supplementary file 1 [file jcm-10-03222-s001.zip › jcm-1250779-conversion/Table S2B.pdf]

Age at onset	Gender	Clinical features	Neuroradiological features	Neurometabolic screening in blood	Skeletal muscle biopsy: histological analysis	Skeletal muscle biopsy: biochemical analysis	Gene	Mutation and level of heteroplasmy
Childhood	F	Dysmorphic features, DD/ID	Cerebral, cerebellar, WM, BG, encephalic trunk involvement	Within the normal limits	Non-specific myopathic signs	COX moderate reduction	MT-CO1	m.6101C>A/p.Ile66Met<br>Heteroplasmy: 40.4% in DNA from skeletal muscle and 16.2% in DNA from peripheral blood
Childhood	M	Psychomotor regression, speech delay/disorder, hypostenia, ataxia/balance disorder, paresis	BG involvement; MRS alterations	Within the normal limits	Non-specific myopathic signs, lipid accumulation, subsarcolemmal rims	CI+CIII moderate reduction;<br>COX moderate reduction;<br>CS increase	MT-ND3	m.10197G>A/p.Ala47Thr<br>Homoplasmy
Childhood	F	Metabolic deompensation, myopathy with hypostenia, DD/ID	Cerebral, WM, BG involvement	n.a.	Atrophic and semi-dystrophic fibers, RRF, complete or partial depletion of oxidative enzymes, COX negative and SDH positive RRF, lipid accumulation, subsarcolemmal rims	CI severe reduction; NADH moderate reduction;<br>CI+CIII severe reduction;<br>SDH moderate reduction;<br>COX severe reduction;<br>CS increase	MTTL1	m.3243A>G<br>Heteroplasmy: 47.6% in DNA from peripheral blood, 42% in DNA from fibroblasts, 71% in DNA from pericardium and 55% in DNA from diaphragm
Infancy	M	Hypotonia, DD/ID	Leigh syndrome	Lactic acid increase	Lipid accumulation, subsarcolemmal rims, non-specific myopathic signs	CI+CIII mild reduction;<br>SDH mild reduction	MT-ND1	m.3697G>A/p.Gly131Ser<br>Homoplasmy
Childhood	F	Dermatological abnormalities, myopathy,	Cerebral involvement; MRS	Lactic acid, Alanine, other metabolites	Semi-dystrophic fibers, subsarcolemmal	CI severe reduction;<br>NADH mild	MTTL2	m.12293G>A<br>Heteroplasmy: 8.7% in DNA from peripheral

		DD/ID	alterations	increase	rims, COX negative fibers	reduction;<br><br>CI+CIII severe reduction;<br><br>SDH severe reduction,<br><br>COX moderate reduction;<br><br>CS increase		blood, 17% in DNA from buccal swab, 47.6% in DNA from urine, and 70% in DNA from skeletal muscle
Childhood	M	Metabolic decompensation, kidney disease, cardiovascular disorder, epilepsy, psychomotor regression, hypostenia, hypotonia, pyramidal signs, hyporeactivity, DD/ID	Cerebral, WM, encephalic trunk involvement	n.a.	Complete or partial depletion of oxidative enzymes, non-specific myopathic signs	Within normal range	MTTC	mnt.5799A>G<br><br>Heteroplasmy: 99% in DNA from skeletal muscle, 29% in DNA from peripheral blood, and 99% in DNA from fibroblasts
Childhood	F	Ataxia, ptosis/extraocular muscle involvement, DD/ID	WM, GB, encephalic trunk	Alanine increase	Lipid accumulation, non-specific myopathic signs	CI severe reduction	MT-ND6	m.14487T>C/p.Met63Val<br><br>Homoplasmy
Childhood	M	Hypotonia, DD, nystagmus, growth failure	Leigh syndrome	Lactic acid and Alanine increase	Insufficient material	Within normal range	MT-ND5	nt.13094T>C/p.Val253Ala<br><br>Heteroplasmy: 57.2% in DNA from skeletal muscle, and 52.9% in DNA from peripheral blood
