# Supplementary material for: The Diagnostic Approach to Mitochondrial Disorders in Children in the Era of Next-Generation Sequencing: A 4-Year Cohort Study
Source: J Clin Med. 2021 Jul 22;10(15):3222. doi: 10.3390/jcm10153222 (PMC8348083; doi:10.3390/jcm10153222)
Supplement: Supplementary file 1 [file jcm-10-03222-s001.zip › jcm-1250779-conversion/Table S3.pdf]

Supplementary Table S3. Variants of unknown significance.

Gene	Mutation	Assessment of heteroplasmy (% of mutated genomes)
mtDNA genes		
MT-CO1	m.6663A>G (p.Ile254Val)	Yes (>98%)
MT-CO3	m.9921G>A (p.Ala239Thr)	Yes (>98%)
MT-CYB	m.15335C>A (p.Leu197Ile)	Yes (>98%)
nDNA genes		
ACACA	c.4032G>T (p.Met1344Ile)/ c.4324C>G (p.Leu1442Val)	not applicable
MFN2	c.1181G>A (p.Arg394His)/ -	not applicable
NDUFA3	c.253_254delTG (p.*85Ser ext*15)/ c.253_254delTG (p.*85Ser ext*15)	not applicable
POLG	c.1156C>T (p.Arg386Cys)/ -	not applicable
POLG	c.803G>C (p.Gly268Ala)/ -	not applicable
POLG	c.752C>T (p.Thr251Ile)/ c.1760C>T(p.Pro587Leu)	not applicable
PPIL1	c.379A>G (p.Thr127Ala)/ c.280+1G>A	not applicable
SETX	c.4660T>G (p.Cys1554Gly)/ -	not applicable
ST3GAL5	c.1166A>G (p.His389Arg)/ c.1024G>A (p.Gly342Ser)	not applicable
SZT2	c.3340G>T (p.Gly1114Trp)/ c.3340G>T (p.Gly1114Trp)	not applicable
VPS13D	c.4022C>T (p.Ser1341Leu)/ c.1696A>G (p.Met566Val)	not applicable
