# Supplementary material for: The Diagnostic Approach to Mitochondrial Disorders in Children in the Era of Next-Generation Sequencing: A 4-Year Cohort Study
Source: J Clin Med. 2021 Jul 22;10(15):3222. doi: 10.3390/jcm10153222 (PMC8348083; doi:10.3390/jcm10153222)
Supplement: Supplementary file 1 [file jcm-10-03222-s001.zip › jcm-1250779-conversion/Table S4.pdf]

	Estimate	z-value	*P* (>|z|)
(Intercept)	-4.1139269	0.008	0.99
Psychomotor regression	-1.80752	-2.300	**0.022**
Epileptic seizures	-0.08718	-0.135	0.89
COX-negative and SDH-positive ragged red fibers	-31.78922	-0.009	0.99
Ragged red fibers and/or ragged blue fibers	14.93034	0.006	0.99
Basal ganglia involvement	-2.24449	-3.366	**0.00076**

Deviance residuals: Min -1.1600, Median -0.4312, Max 2.2190.
